# Palliative care in the emergency department: an educational investigation and intervention

**DOI:** 10.1186/s12904-018-0293-5

**Published:** 2018-03-07

**Authors:** Jessica M. Goldonowicz, Michael S. Runyon, Mark J. Bullard

**Affiliations:** 1 Department of Emergency Medicine, Carolinas Medical Center, Carolinas Healthcare System, 1000 Blythe Blvd., 3rd Floor MEB, Charlotte, NC 28203 USA; 2 Carolinas Simulation Center, Carolinas Healthcare System, Charlotte, USA

## Abstract

**Background:**

To investigate the value of a novel simulation-based palliative care educational intervention within an emergency medicine (EM) residency curriculum.

**Methods:**

A palliative care scenario was designed and implemented in the simulation program at an urban academic emergency department (ED) with a 3-year EM residency program. EM residents attended one of eight high-fidelity simulation sessions, in groups of 5–6. A standardized participant portrayed the patient’s family member. One resident from each session managed the scenario while the others observed. A 45-min debriefing session and small group discussion followed the scenario, facilitated by an EM simulation faculty member and a resident investigator. Best practices in palliative care were highlighted along with focused learner performance feedback. Participants completed an anonymous pre/post education intervention survey.

**Results:**

Forty of 42 EM residents (95%) participated in the study. Confidence in implementing palliative care skills and perceived importance of palliative care improved after this educational intervention. Specifically, residents 1) felt EM physicians had an important role in palliative care, 2) had increased confidence in the ability to determine patient decision-making capacity, 3) had improved confidence in initiating palliative discussions/treatment, 4) believed palliative education was important in residency, and 5) felt simulation was an effective means to learn palliative care. Differences noted between PGY1 and PGY 3 training levels in survey responses disappeared post-intervention. Residents noted being most comfortable with delivering bad news and symptom management and least comfortable with disease prognostication. Residents reported time constraints and implementation logistics in the ED as the most challenging factors for palliative care initiation.

**Conclusion:**

Our case-based simulation intervention was associated with an increase in both the perceived importance of ED palliative care and self-reported confidence in implementing palliative care skills. Time constraints and implementation logistics were rated as the most challenging factors for palliative care initiation in the ED.

**Electronic supplementary material:**

The online version of this article (10.1186/s12904-018-0293-5) contains supplementary material, which is available to authorized users.

## Background

Palliative medicine has been practiced for centuries; however, only recently has it been formally recognized as a medical subspecialty in the United Sates. In 2014, the Institute of Medicine’s (IOM) Report, *Dying in America: Improving Quality and Honoring Individual Preferences Near the End of Life,* urged that palliative care be considered as core training for every clinician who cares for seriously ill patients nearing the end of life [1]. Additionally, the IOM emphasized the importance of effective communication within the field of palliative care and highlighted initiatives to foster competencies within medical curricula [1].

The specialty of emergency medicine has traditionally focused on resuscitation, stabilization, and management of acute disease processes, with definitive and end-of-life care provided by other medical specialties. Some might argue that palliative medicine represents non-emergent interventions; however, palliative care has many varying definitions. The American Academy of Hospice and Palliative Medicine defines palliative medicine as a means to “…prevent and relieve suffering and to support the best possible quality of life for patients facing life-threatening or debilitating illness…regardless of the stage of the disease or the need for other therapies” [2]. The World Health Organization defines palliative care as “…an approach that improves the quality of life of patients and their families facing the problems associated with life threatening illness through the prevention and relief of suffering by early identification, impeccable assessment and treatment of pain and other problems, physical, psychosocial, and spiritual” [3]. Based upon these descriptions, palliative and emergency medicine should not be mutually exclusive but rather synergistic, and their relationship must evolve in order to achieve a common purpose of providing complex, comprehensive, and compassionate care [4].

Despite this common purpose, emergency physicians face many challenges in delivering effective palliative care. Appropriate decisions for palliative care are dependent on accurately prognosticating a patient’s disease process, and predicting impending mortality can be challenging. Physicians often hesitate when discussing prognosis due to: not being confident with the diagnosis, concerns of diminishing patients’ hope, or believing that patients are not prepared to hear forthcoming information [5]. Subsequently, aggressive interventions are often initiated as result of uncertainty, time constraints, illness complexity, and medico-legal threats [5, 6]. Due to this, interventions may ultimately be misaligned with overall goals of care, and retrospectively viewed as futile, harmful, or painful [7]. In addition, crowded spaces, a noisy environment, frequent interruptions, and compromised privacy make initiation of palliative care discussions less than ideal in the emergency setting [5]. In order for physicians to have impactful patient and family interactions, physicians must be able to provide honest, timely information, build rapport, and affirm patient wishes through thoughtful discussion of futile interventions, which may not be feasible based upon the above situational constraints.

It has been suggested that brief communication workshops can provide physicians with practical skills that can be applied immediately to improve the success of challenging palliative care discussions [8, 9]. It is also well recognized that the learning experiences that residents receive during their training establish the foundation for future behaviors, attitudes, and methods of practice for the remainder of their professional careers [10]. As such, we developed and implemented an educational intervention aimed at improving education on palliative care medicine. Additionally, we aimed to evaluate attitudes and impressions regarding palliative educational training. The specific goal of this study was to examine the perceived value of simulation-based palliative care education within an emergency medicine residency program where no previous palliative care educational components existed. Additionally, we sought to evaluate the need for increased emphasis and development of further palliative care curricula to meet our resident learner’s needs.

## Methods

This was a prospective study of a simulation-based palliative care educational intervention. The study protocol was reviewed by the Carolinas HealthCare Institutional Review Board and deemed exempt as research involving anonymous educational surveys obtained as part of normal education practice. The study was conducted at the Carolinas Simulation Center, a multi-disciplinary facility accredited by both the American College of Surgeons as a Comprehensive Education Institute and by the Society for Simulation in Healthcare. Founded in 1976, the Carolinas Medical Center Emergency Medicine Residency Program is a post-graduate year (PGY) 1–3 program with 14 residents per class.

As a component of the current emergency medicine simulation program, a palliative care scenario was designed, implemented, and studied. This same scenario was presented in eight, 1 h palliative care simulation sessions conducted during this educational intervention. One resident per session was chosen at random from each small group of 5–6 resident learners during each session. This resident participated in a simulation case with a high-fidelity mannequin and standardized participant who portrayed the patient’s daughter. The remainder of learners observed the encounter via live streaming video.

Given previously cited challenges in accurate prognostication of a patient’s disease process and its implication on palliation, investigators created an uncomplicated case scenario with a patient having an obvious end-of-life disease trajectory. The case was designed so that learners would be more likely to focus on palliative issues rather than emergent resuscitation and stabilization. The simulated scenario involved an elderly male patient with history of dementia, chronic obstructive pulmonary disease, and recently diagnosed pancreatic cancer. The patient presented to the emergency department with hypoxia, unresponsive to non-invasive oxygen supplementation, worsening altered mental status and was without decision-making capacity. No advanced directives were clearly defined. The learner was expected to appropriately care for the critically ill patient while discussing with the family member the current disease state and treatment goals; including consideration of intubation versus palliative care interventions.

Following each case, an approximate 45-min shared debriefing session, consisting of the scenario participant and observers took place to enforce current best practices in emergency palliative care. At least one of three EM simulation faculty members specifically trained in simulation debriefing and the resident investigator facilitated each debriefing. Facilitator dyads consisted of the EM simulation faculty as a lead-debriefer and the resident investigator serving as an associate debriefer [11]. This debriefing methodology assured that all debriefings were uniform.

Predetermined educational points were highlighted during the debriefing and included: providing an honest prognosis to the best of one’s ability, taking care to honor the patient’s wishes/values regarding treatment goals, communicating in straightforward language with empathy, and building trust with the patient and family. In particular, A Rapid Palliative Care Assessment Tool, developed as part of the Education in Palliative and End-of-life Care (EPEC) Project Curriculum (Table 1), was included in each educational feedback session [9, 12]. Additionally, discussion regarding advanced directives and local law was incorporated into each session.

**Table 1:** Rapid Palliative Care Assessment- A Secondary Survey of ABCDs (Emanuel)

A-Advance directives	Any documents in place detailing wishes for life-sustaining measures?
B-Better	How can you help the patient feel better? Manage symptoms during acute resuscitation while determining the appropriate degree of resuscitative measures for the situation.
C-Caregivers	Is there anyone present at the bedside, in the waiting area, or who can be reached by phone? Consider their needs and desires.
D-Decision making capacity	Can the patient make their own decision regarding their care?

We conducted paired, anonymous, convenience sample surveys of emergency medicine residents at a single academic, urban tertiary care hospital prior to and immediately following a simulation-based educational intervention (Additional file 1). While the simulation-based palliative care case was mandatory for all 42 residents within residency program, participation in the survey study was voluntary. There were no exclusion criteria, with the exception of the resident investigator. None of the simulation faculty served in any formal evaluative roles within the residency. Study participants were randomly assigned anonymous numerical identifiers and surveys containing seven identical questions were paired for pre- and post-intervention analysis. The questions aimed to evaluate: 1) perceived opportunities for/need of palliative care education, 2) perceptions of palliative care and the emergency physician’s role, 3) comfort in understanding palliative care domains and initiating discussions with patients and families, 4) confidence in determining patient decision making capacity, and 5) attitudes towards different learning modalities. Participants marked their response to each survey question on a 100 mm visual analog scale.

Categorical data were summarized as counts and percentages and continuous variables as medians and interquartile ranges (IQRs) and differences with 95% confidence intervals (CIs). Pre- and post-intervention survey responses were compared using the Wilcoxon signed ranks test and a Bonferroni-adjusted *p*-value threshold of 0.007 was defined as a conservative estimate of statistical significance. We also present the median differences and associated 95% confidence interval for each survey item. Comparisons of responses by postgraduate year (PGY) were made with the Kruskal-Wallis test with post-hoc pairwise comparisons with the Dwass-Steel-Critchlow-Fligner test, where appropriate. All analyses were performed with StatsDirect, version 3.0.167 (StatsDirect Ltd., Cheshire, UK).

## Results

**Fig. 1:**
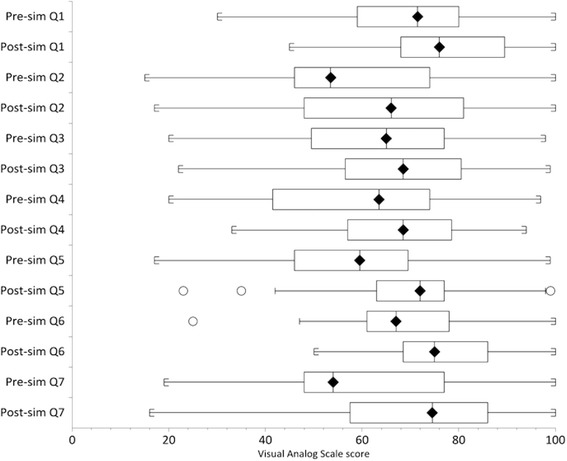
Pre/post survey responses for all PGY

Forty of 42 possible EM residents (95%) participated in the study. One resident was out of the country during the intervention and one resident was excluded from participation, as she was a study investigator. Descriptive statistics of study participants were: 1) Gender: 23 male/17 females, 2) Ethnicity: 38 Caucasian/1 Asian/1 African American, 3) Age: mean 30.225 years/range 25–40 years. Residents’ confidence in implementing palliative care skills and the perceived importance of palliative care was significantly improved after this educational intervention (Fig. 1). Specifically, following the intervention, EM residents rated the following statements higher compared with the pre-intervention survey: 1) The role of the emergency medicine physician in palliative care is important (*p* = 0.0003), 2) I feel confident in my ability to determine a patients decision-making capacity (*p* = 0.0005), 3) I feel confident with initiating palliative care discussions and treatment in the emergency department (*p* <  0.0001), 4) Palliative care education is an important component to my residency training (*p* = 0.0013), and 5) Simulation is an effective educational tool to learn palliative care skills (*p* <  0.0001) (Table 2).

**Table 2:** Pre/post survey responses for all PGY

Question	Pre-sim median (IQR)	Post-sim median (IQR)	Median difference (95% CI)	*P*-value
1) The role of the emergency medicine physician in palliative care is important.	71.5 (59–80)	76 (68–89.5)	+ 7.5 (3.5–11)	0.0003
2) If I suspect a high risk of morbidity and/or mortality during an emergency department encounter, how often do I have this discussion with my patient?	53.5 (46–74)	66 (48–81)	+ 3 (− 0.5–8.5)	0.0792
3) I feel confident in my understanding of palliative care.	65.0 (49.5–77)	68.5 (56.5–80.5)	+ 4 (− 1–11)	0.1265
4) I feel confident in my ability to determine a patient’s decision-making capacity.	63.5 (41.5–74)	68.5 (57–78.5)	+ 7 (2.5–12)	0.0005
5) I feel confident with initiating palliative care discussions and treatment in the emergency department.	59.5 (46–69.5)	72 (63–77)	+ 11 (5.5–17)	< 0.0001
6) Palliative care education is an important component to my residency training.	67 (61–78)	75 (68.5–86)	+ 6.5 (2.5–10.5)	0.0013
7) Simulation is an effective educational tool to learn palliative care skills.	54 (48–77)	74.5 (57.5–86)	+ 9.5 (5–14)	< 0.0001

*PGY* post-graduate year, *IQR* interquartile range, *CI* confidence interval

When the survey responses were analyzed by PGY year, there were significant differences between PGY-1 and PGY-3 ratings on five of the seven items on the pre-intervention survey and all but two of these differences disappeared on the post-intervention survey. The two items with persistent differences on the post-intervention survey were: “If I suspect a high risk of morbidity and/or mortality during and emergency department encounter, how often do I have this discussion with my patient?” and “I feel confident with initiating palliative care discussions and treatment in the emergency department.” Both items were rated significantly lower by the PGY-1 residents than by the PGY-3 residents (*p* = 0.020 and 0.019, respectively). There was one significant difference between the PGY-1 and PGY-2 responses on the pre-intervention survey that disappeared in the follow-up survey. This item was: “I feel confident with initiating palliative care discussions and treatment in the emergency department.” Likewise, there was one significant difference between the PGY-2 and PGY-3 responses on the pre-intervention survey that disappeared in the follow-up survey. This item was: “Palliative care education is an important component to residency training.” Finally, there was one significant difference between the PGY-2 and PGY-3 groups on the post-intervention survey that was not seen on the pre-intervention survey. This item was: “If I suspect a high risk of morbidity and/or mortality during an emergency department encounter, how often do I have this discussion with my patient?” While both groups rated the item higher post-intervention, the PGY-3 group did so out of proportion to the PGY-2 participants (Tables 3 and 4).

**Table 3:** Post-hoc pairwise comparisons of responses by postgraduate year (PGY) with representative *p*-values

Question	Pre-sim PGY-1 vs 2	Pre-sim PGY-1 vs 3	Pre-sim PGY-2 vs 3	Post-sim PGY-1 vs 2	Post-sim PGY-1 vs 3	Post-sim PGY-2 vs 3
1	0.9364	0.0291	0.0502	–	–	–
2	0.5407	0.0202	0.1527	0.9858	0.0203	0.022
3	–	–	–	–	–	–
4	0.1067	0.0412	0.5254	–	–	–
5	0.0025	0.0044	0.4322	0.0658	0.0189	0.4935
6	0.9365	0.0384	0.0233	–	–	–
7	–	–	–	–	–	–

**Table 4:** Median and IQR by PGY (PGY 1 *N* = 14; PGY 2 *N* = 14; PGY 3 *N* = 12)

Question	Pre-sim median (IQR) PGY-1	Pre-sim median (IQR) PGY-2	Pre-sim median (IQR) PGY-3	Post-sim median (IQR) PGY-1	Post-sim median (IQR) PGY-2	Post-sim median (IQR) PGY-3
1	63 (51–73)	61 (59–77)	78 (73.5–96)	76 (72–89)	74 (68–81)	82.5 (68–96)
2	47 (28–68)	59 (46–72)	75 (52–95.5)	58.5 (40–72)	64 (46–70)	81 (70–94.5)
3	56.5 (27–77)	64.5 (59–77)	68.5 (61–89.5)	64.5 (58–81)	63 (50–74)	77.5 (66.5–83.5)
4	43.5 (28–65)	66 (59–68)	71 (60.5–89)	61.5 (51–72)	72.5 (56–78)	75 (63.5–84.5)
5	43.5 (22–49)	64 (58–69)	69.5 (55.5–86)	64.5 (47–72)	73.5 (68–75)	77 (70.5–81.5)
6	65 (60–71)	64 (57–70)	84 (67.5–99)	74.5 (67–80)	74.5 (70–80)	83 (71–96)
7	70.5 (48–83)	52 (50–55)	67 (47–83.5)	73.5 (56–82)	70.5 (48–80)	79 (62.5–88)

*PGY* post-graduate year, *IQR* interquartile range

Overall, residents noted being most comfortable with delivering bad news and symptom management and least comfortable with disease prognostication. Residents additionally reported that time constraints and implementation logistics in the emergency department as most challenging factors for palliative care initiation. Residents rated bedside teaching as the best method of learning palliative care skills, followed by small group learning, simulation, the lecture format, and online asynchronous modules (Table 5).

**Table 5:** Learning methods

What is the best method of learning palliative care? Mean rating for learners (1 = best, 5 = least)	
Bedside teaching	1.55
Small group learning	1.94
Simulation	2.31
Lecture format	3.42
Online asynchronous module	4.10

## Discussion

Our case-based simulation intervention was associated with a significant increase in both the perceived importance of ED palliative care and self-reported confidence in implementing palliative care skills among the 40-participating emergency medicine resident physicians. When asked their preferences for education content delivery, participants rated small group learning and simulation, both of which are elements of our intervention, as preferable to lectures or online asynchronous modules. Time constraints and implementation logistics were rated as the most challenging factors for palliative care initiation in the ED.

Our study echoed previous literature in that it highlighted a need for palliative care educational curricula in medical education and specifically within EM residency training [8, 13–15]. Due to advances in medicine, patients are living longer with chronic complex diseases, thus creating an increasing need for common knowledge in palliative care medicine. While recent medical literature reflects a heightened interest in the field of palliative care [6], there remains a paucity of educational interventions; particularly within the emergency department setting [13] despite the fact that a majority of critically-ill patients are being admitted to the hospital through the emergency department. Lack of education, knowledge, and comfort have been cited by emergency clinicians as barriers to providing effective palliative care in the emergency department; however, it is often in this unpredictable setting that trajectories for continued care are established [16]. By incorporating palliative care skill sets into daily practice, emergency medicine physicians have the unique opportunity to make pivotal decisions, not only for managing acute symptoms but also for establishing the groundwork for true patient-centered care [16].

Simulation-based education was chosen for this educational intervention due to its active and experiential components. Both medical and non-medical literature clearly suggests that simulation improves knowledge and skill acquisition while creating an educational method with a high level of learner satisfaction [17]. Medical simulation is a unique educational modality because learners are able to practice procedures, pharmaceutical interventions, as well as develop complex ethical and spiritual communication skills prior to real-life encounters with patients. Additionally, active learning methods have been demonstrated to be superior to traditional lecture-based learning formats in student retention of information for knowledge transfer and attitude change [18]. Our learners also confirmed these previous beliefs. While learners rated bedside teaching as the best method of learning palliative care skills, the next most preferred methods were small group learning and simulation, both of which were elements of our intervention and rated as more preferable than the traditional lecture format or online asynchronous modules. This is likely due to the significant interpersonal skills that are integral to palliative care that do not translate as well to the lecture or asynchronous learning formats.

The results from the surveys obtained before and after our educational intervention provided beneficial insight into the attitudes and experiences of our EM residents regarding palliative medicine. Learners identified palliative care as an important component to emergency medicine training and practice; however, a more comprehensive knowledge of the specialty’s capabilities and limitations would require further education. Residents were most comfortable with breaking bad news and least comfortable with prognostication, correlating with previous educational experiences during their clinical training that focused on facilitating difficult conversations such as death notification, but lacked more comprehensive palliative care skill development. Prior to the educational intervention, 63% of participants reported five or less palliative care learning experiences during their residency with post-graduate training year not affecting responses.

When comparing responses by PGY, it is noteworthy that significant differences existed between training levels, such as resident perspectives regarding palliative medicine being important to the emergency physician, confidence in the ability to determine patient decision-making capacity, and palliative care education as an important component to residency training. These disparities in response disappeared post educational intervention. The significant post-interventional changes in response noted between PGY 1 and PGY 3 training levels best represent an increased PGY 1 confidence and awareness of the importance of palliative care to the field of emergency medicine and to EM residency training, as median responses by PGY increased significantly in each of these domains (Table 4).

### Limitations

Our sample size was small and limited to a single emergency medicine residency program and the comparisons of responses by PGY were even more limited by the number of participants at each level. Consent was obtained and surveys were completed on the same day as the educational intervention. While an in-person method of subject acquisition led to robust participation by eligible learners, it must be considered whether participants were primed to think and/or act in a certain way during the simulation session. Although there were four other unrelated simulation scenarios during each session, learners may have handled the experience differently had it not been foreshadowed that palliative care was one of the simulation topics of the day.

Due to the anonymous nature of our survey, we were unable to compare the results of the residents who actively managed the simulation case to those who simply observed, however, limited studies have demonstrated the benefit of simulation observation [19, 20]. Additionally, one of the authors recently completed an yet unpublished study that demonstrated no advantage of one learner role (participant vs. observer) in simulation-based education when paired with high quality debriefing.

Post-intervention responses may have differed if surveys were administered at a different time interval, understanding that the exact time period for interval assessment of simulation-based learning is still unknown. Skill and knowledge can quickly decay after simulation-based education [21–24], with one study reporting decay as early as 2 weeks after simulation-based education [24]. The authors chose to collect data from learner participants immediately after the simulation experience while the learners’ reactions were still dynamic. Additionally, not all individuals learn or process information similarly. For some, taking additional time for reflection or real-life implementation of the knowledge and skills acquired through the intervention may have changed the response outcomes. For others, attitudes regarding goals of care in the emergency department primarily focus on resuscitation and disposition, and integration of palliative measures may not be regarded as practical or valuable within the acute care setting. Unfortunately, this study did not evaluate validity and reliability of the survey instrument, and also did not have a follow up assessment after the index survey, which may have affected results and unfortunately cannot be extrapolated to long-term benefits of the education.

Finally, whether increased confidence equates to real clinical practice remains unknown. This study was designed to evaluate the perceived value of a simulation-based educational intervention and subsequent learner confidence in palliative care topics and unfortunately may not reflect changes in real life practice. However, survey feedback demonstrated considerable increases in learner confidence in palliation topics including resident confidence in implementing palliative care in real clinical settings.

## Conclusions

Our case-based simulation intervention was associated with an increase in both the perceived importance of ED palliative care and self-reported confidence in implementing palliative care skills. Participants rated small group learning and simulation, both of which are elements of our intervention, as preferable to lectures or online asynchronous modules. Time constraints and implementation logistics were rated as the most challenging factors for palliative care initiation in the ED.

## Additional files

Additional file 1: Pre/Post Simulation Intervention Survey Questions. (DOCX 80 kb)

## Abbreviations

CI: Confidence interval
ED: Emergency department
EM: Emergency medicine
EPEC: Education in palliative and end-of-life care project curriculum
IOM: Institute of Medicine
IQR: Interquartile range
PGY: Post-graduate year
