# Supplementary material for: Palliative care in the emergency department: an educational investigation and intervention
Source: BMC Palliat Care. 2018 Mar 7;17:43. doi: 10.1186/s12904-018-0293-5 (PMC5842635; doi:10.1186/s12904-018-0293-5)
Supplement: Supplementary file 1 — Pre/Post Simulation Intervention Survey Questions. (DOCX 80 kb) [file 12904_2018_293_MOESM1_ESM.docx]

**Appendix A**

**Pre- Simulation Intervention Survey Questions**

How do you define palliative care?

Comfort care measures only

Do Not Resuscitate/Do Not Intubate

Focus on symptom management

Patient has a terminal illness and 6 months or less to live

Managing imminent death

The role of the emergency medicine physician in palliative care is important.

[Longitudinal survey 0 -100 – 0 = strongly disagree, 100 = strongly agree]

If I suspect a high risk of morbidity and/or mortality during an emergency department encounter, how often do I have this discussion with my patient?

[Longitudinal survey 0 -100 – 0 = never, 100 = always]

I feel confident in my understanding of palliative care.

[Longitudinal survey 0 -100 – 0 = strongly disagree, 100 = strongly agree]

I feel confident in my ability to determine a patient’s decision making capacity.

[Longitudinal survey 0 -100 – 0 = strongly disagree, 100 = strongly agree]

I feel confident with initiating palliative care discussions and treatment in the emergency department

[Longitudinal survey 0 -100 – 0 = strongly disagree, 100 = strongly agree]

Palliative Care education is a needed component to my residency training

[Longitudinal survey 0 -100 – 0 = strongly disagree, 100 = strongly agree]

How many lectures (informal/bedside and/or formal/presentations) on palliative care have you had during your residency training?

[Provide a numerical answer 0-10, 10-20, 20-30, 30-40, 40-50, 50+]

What are your biggest concerns regarding palliative care in the emergency department? (Rank in order 1 = most, 5 = least)

Logistics of implementation

Medico-legal consequences

Moral/ethical/religious considerations

Scope of practice

Time constraints

What is the best method of learning palliative care?

(Rank in order 1= best, 5 = worst)

Bedside teaching in ED

Lecture style presentation

Online e-learning asynchronous modules

Simulation cases

Small group learning

**Post-Simulation Intervention Survey**

The role of the emergency medicine physician in palliative care is important.

[Longitudinal survey 0 -100 – 0 = strongly disagree, 100 = strongly agree]

If I suspect a high risk of morbidity and/or mortality during an emergency department encounter, how often do I have this discussion with my patient?

[Longitudinal survey 0 -100 – 0 = never, 100 = always]

I feel confident in my understanding of palliative care.

[Longitudinal survey 0 -100 – 0 = strongly disagree, 100 = strongly agree]

I feel confident in my ability to determine a patient’s decision making capacity.

[Longitudinal survey 0 -100 – 0 = strongly disagree, 100 = strongly agree]

I feel confident with initiating palliative care discussions and treatment in the emergency department

[Longitudinal survey 0 -100 – 0 = strongly disagree, 100 = strongly agree]

Palliative Care education is a needed component to my residency training

[Longitudinal survey 0 -100 – 0 = strongly disagree, 100 = strongly agree]

My confidence in providing palliative care measures has changed following simulation participation

[Longitudinal survey 0 -100 – 0 = strongly disagree, 100 = strongly agree]

Simulation is an effective educational tool to learn palliative care skills

[Longitudinal survey 0 -100 – 0 = strongly disagree, 100 = strongly agree]

A greater focus on palliative care education is needed within our residency curriculum

[Longitudinal survey 0 -100 – 0 = strongly disagree, 100 = strongly agree]

In which domains of palliative care do you feel the most knowledgeable and comfortable?

(Rank in order 1 = most, 5 = least)

Breaking bad news

Discussing code status

Discussing palliative care approach with patient/family

Pain/Symptom management

Prognostication
